# Family matters in unraveling human longevity

**DOI:** 10.18632/aging.104218

**Published:** 2020-11-28

**Authors:** Niels van den Berg

**Affiliations:** 1 Department of Biomedical Data Sciences, section of Molecular Epidemiology, Leiden University Medical Center, 2333 ZA, Leiden, the Netherlands

**Keywords:** aging, longevity, social, family, genes, familial longevity

The human life expectancy has doubled over the past 200 years in most industrialized countries. However, even though people started to live longer, the time spent in good physical and cognitive health is considerably short. In fact, 70% of the 65 year old people and 90% of the 85 year old people already have at least one disease and on average four diseases [1]. In contrast to the general population, a few remarkable persons become exceptionally old without the burden of age related diseases (e.g. high blood pressure, malignancies, and type 2 diabetes) [2]. Hence, the study of such exceptionally old individuals (longevity) is important as they likely harbor gene-environment interactions which beneficially regulate molecular pathways involved in longevity, resistance to disease, resilience to negative side-effects of treatment and therefore healthy aging.

Most knowledge on regulators of aging and longevity has been obtained from animal models in the laboratory. Such models revealed nine key mechanisms (hallmarks of aging), and more specifically, glucose and fat metabolism pathways to be involved in ageing and longevity [3]. The study of human longevity, however, is more complex than in animals, as human genomes and lives are extremely heterogenous. As such, both genetic and non-genetic factors (e.g. lifestyle, environment, and social network), as well as their interactions are involved in human longevity. It has been estimated that age at death (lifespan) attributes for ~25% to genetic variation and this number rises for long-lived individuals as shown by its strong familial clustering [4,5]. Nevertheless, two decades of genetic research to understand the mechanisms of longevity and healthy aging had limited robust results. Amongst a number of potential determinants, only the *APOE* and *FOXO3A* genes have been consistently identified [6].

One of the main reasons for the difficulty of identifying genes promoting longevity and healthy aging is the lack of a consistent definition for heritable longevity, which resulted in a mix of sporadically long-lived cases with those descending from a long-lived family and a large variation of longevity definitions used in longevity research [4,5]. The presence of sporadically long-lived cases is illustrated by the increase of centenarians in the United States between 1994 and 2012 from 1 in 10,000 to 2 in 10,000. Secondly, Genome Wide Association (GWA) analysis, the leading method in complex disease mapping, relies on comparing living long-lived individuals (cases) with averagely living individuals (controls). These averagely lived individuals can in fact become long-lived over time, thus potentially confusing cases and controls. In addition, recent research revealed the importance of rare and structural variants [7] in addition to the common single nucleotide polymorphisms (SNPs) studied in GWAS. Thirdly, socio-behavioral and environmental factors, such as lifestyle, socio-economic status, social network, and the living environment shaped the aging process of long-lived persons in interaction with their genes [8]. However, these factors are rarely included in genetic longevity studies and surprisingly little is known about how they cluster in long-lived families.

The issue of unintentionally including sporadically long-lived cases has recently been addressed in two studies, using multiple large scale family tree databases; the Utah Population Database (UPDB), the LINKing System for historical family reconstruction (LINKS), and the Historical Sample of the Netherlands Long Lives (HSN-LL) which contain thousands of families [4,5]. The studies showed that longevity is only transmitted across generations if at least 30% of the ancestors of a person belonged to the top 10% survivors of their birth cohort and the persons themselves also belong to the 10% longest lived. Importantly, 27% of the HSN-LL research persons showed a survival pattern similar to the general population even though they had at least one long-lived parent. Based on these results, the Longevity Relatives Count (LRC) score was developed as an instrument to identify genetically enriched long-lived persons for case inclusion in genetic studies and thus avoid the inclusion of sporadically long-lived persons [5].

**Figure 1:**
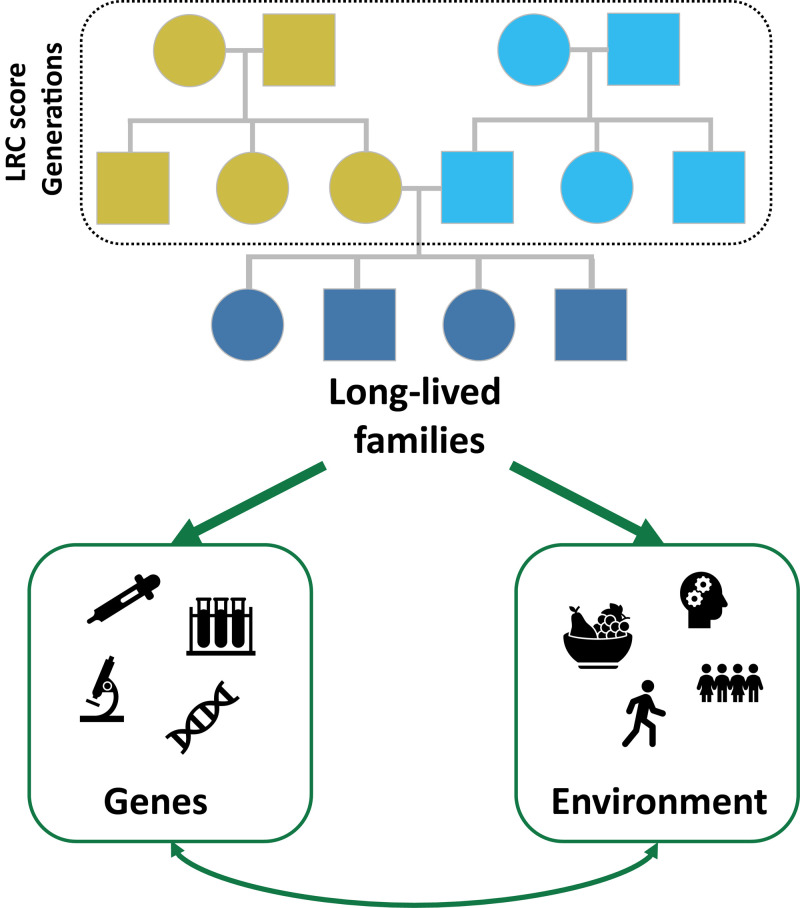
Long-lived families, Identified using the Longevity Relatives Count (LRC) score, provide a unique opportunity to investigate the cohesion and interplay between genes and socio-behavioral and environmental aspects (G x E interactions). Squares are males and rounds are females. Light blue colors represent the paternal ancestors and yellow colors represent the maternal ancestors. Dark blue colors represent potential study participants. Both maternal as paternal ancestral information can be used to calculate the LRC score and identify long-lived families, thus reducing the number of sporadically long-lived persons.

Population-wide family tree data (e.g. LINKS, the UPDB, and platforms such as My Heritage) may be used to address the remaining issues and moreover, are becoming increasingly available. For example, the Netherlands is currently linking all civil certificates into nation-wide multigenerational family trees and a similar initiative is ongoing in Denmark. This increasing availability of population-wide family tree data makes it possible to extend our current perspective, which is mainly focused on (long-lived) individuals, to long-lived families by applying the LRC score (Figure 1). Such long-lived families provide the unique opportunity to investigate the cohesion and interplay between genes and socio-behavioral and environmental components (G x E interactions). To increase our understanding of the mechanisms of longevity and healthy aging it is therefore important for epidemiological studies with existing molecular data to integrate a family dimension by collecting genealogical, socio-behavioral and environmental data. These epidemiological and other family based studies can recruit living members from heritable longevity families and collect relevant epidemiological and socio-behavioral data. Genetic analyses should then include the study of rare and structural ‘private’ variants in addition to common variants [7]. Additionally, studies based on living study participants who have not yet reached the ages to express longevity, but have ancestral survival data, such as UK Biobank, may now better distinguish cases by incorporating a liability based on the LRC score. To summarize, the ultimate mechanistic insight for human longevity is expected from genetic studies which have a significantly increased chance of success if the right definitions, data, and non-genetic (e.g. socio-behavioral) covariates and confounders are taken into account.
